# Population structure and genetic diversity characterization of a sunflower association mapping population using SSR and SNP markers

**DOI:** 10.1186/s12870-014-0360-x

**Published:** 2015-02-13

**Authors:** Carla V Filippi, Natalia Aguirre, Juan G Rivas, Jeremias Zubrzycki, Andrea Puebla, Diego Cordes, Maria V Moreno, Corina M Fusari, Daniel Alvarez, Ruth A Heinz, Horacio E Hopp, Norma B Paniego, Veronica V Lia

**Affiliations:** Instituto de Biotecnología, Centro de Investigaciones en Ciencias Veterinarias y Agronómicas (CICVyA), Instituto Nacional de Tecnología Agropecuaria (INTA), Nicolás Repetto y Los Reseros s/n (1686), Hurlingham, Buenos Aires Argentina; Consejo Nacional de Investigaciones Científicas y Técnicas–CONICET, Saavedra 15, C1083ACA Ciudad Autónoma de Buenos Aires, Argentina; Facultad de Ciencias Exactas y Naturales, Universidad de Buenos Aires, Pabellón 2, Ciudad Universitaria (1428), Buenos Aires, Argentina; Estación Experimental Agropecuaria Manfredi, Ruta Nac. nro. 9 km 636 (5988), Manfredi, Córdoba (INTA) Argentina; Currently at System Regulation Group, Metabolic Networks Department, Max Planck Institute of Molecular Plant Physiology, Am Mühlemberg 1, D-14476 Potsdam-Golm, Germany

**Keywords:** Sunflower breeding, Genetic resources, SNP, SSR, Association mapping

## Abstract

**Background:**

Argentina has a long tradition of sunflower breeding, and its germplasm is a valuable genetic resource worldwide. However, knowledge of the genetic constitution and variability levels of the Argentinean germplasm is still scarce, rendering the global map of cultivated sunflower diversity incomplete. In this study, 42 microsatellite loci and 384 single nucleotide polymorphisms (SNPs) were used to characterize the first association mapping population used for quantitative trait loci mapping in sunflower, along with a selection of allied open-pollinated and composite populations from the germplasm bank of the National Institute of Agricultural Technology of Argentina. The ability of different kinds of markers to assess genetic diversity and population structure was also evaluated.

**Results:**

The analysis of polymorphism in the set of sunflower accessions studied here showed that both the microsatellites and SNP markers were informative for germplasm characterization, although to different extents. In general, the estimates of genetic variability were moderate. The average genetic diversity, as quantified by the expected heterozygosity, was 0.52 for SSR loci and 0.29 for SNPs. Within SSR markers, those derived from non-coding regions were able to capture higher levels of diversity than EST-SSR. A significant correlation was found between SSR and SNP- based genetic distances among accessions. Bayesian and multivariate methods were used to infer population structure. Evidence for the existence of three different genetic groups was found consistently across data sets (*i.e*., SSR, SNP and SSR + SNP), with the maintainer/restorer status being the most prevalent characteristic associated with group delimitation.

**Conclusion:**

The present study constitutes the first report comparing the performance of SSR and SNP markers for population genetics analysis in cultivated sunflower. We show that the SSR and SNP panels examined here, either used separately or in conjunction, allowed consistent estimations of genetic diversity and population structure in sunflower breeding materials. The generated knowledge about the levels of diversity and population structure of sunflower germplasm is an important contribution to this crop breeding and conservation.

**Electronic supplementary material:**

The online version of this article (doi:10.1186/s12870-014-0360-x) contains supplementary material, which is available to authorized users.

## Background

Cultivated sunflower (*Helianthus annuus* L. var. macrocarpus) is one of the most important oilseed crops, with a cultivated area of 25 million hectares worldwide (www.sunflowernsa.com). Its annual production ascends to 36 million metric tons and it is mainly concentrated in the Russian Federation, Ukraine, European Union, and Argentina, which is the fourth largest producer and the third oil exporter [1].

The history of introduction and adaptation of sunflower in Argentina is closely related to that of the human migration flows. The crop first arrived via Jewish immigrants bringing small quantities of seeds from the south of Russia. After that, the introduction of early materials from Russia, Canada and Romania, as well as the introgression with wild Helianthus species allowed the emergence of the Argentinean germplasm, which has a distinct genetic constitution and is well adapted to local growing conditions [2,3].

Since its domestication by pre-Columbian civilizations, sunflower has long been the focus of breeding efforts. The introduction of heterosis, first described in 1966 [4], the incorporation of cytoplasmic male sterility after interspecific crossing with *H. petiolaris* Nutt [5], and the development of fertility restorer lines by Kinman in 1970 [6] allowed practical development of sunflower hybrids, with higher yield and quality potential, high homogeneity, maturing time synchronicity and better adaptation to field applications [7].

Despite the optimism for continued improvement by conventional breeding, the need to increase efficiency and precision, and save time, resources and efforts, has motivated the application of new breeding strategies based on genetics. Association mapping (AM) is a relatively recent quantitative trait loci (QTL) mapping approach, that has the potential for resolution to the level of individual genes (alleles) [8]. In contrast to classical QTL mapping techniques used in the analysis of complex traits, AM is a method that detects relationships between phenotypic variation and gene polymorphisms in existing germplasm collections, without development of mapping populations [9,10]. Until now, only four AM studies have been reported for sunflower. The first one was conducted by Fusari et al. [9] using a set of inbred lines from the breeding program of the National Institute of Agricultural Technology (INTA, Argentina), whereas the remaining three were based on germplasm collections from the USDA North Central Regional Plant Introduction Station (NCRPIS), the French National Institute for Agricultural Research, INRA and the USDA-ARS, Northern Crop Science Laboratory [11-13].

The genetic diversity and population structure of North American and European resources has been exhaustively assessed by Coque et al. [11] and Mandel et al. [12]. In contrast, knowledge of the genetic constitution and variability levels of the Argentinean AM population is still scarce, rendering the worldwide diversity map of cultivated sunflower incomplete. Different kinds of molecular markers are available for sunflower, with microsatellites (single sequence repeats, SSR) and single nucleotide polymorphisms (SNP) being the most popular. More than 2000 SSR have been developed from genomic (gSSR) and EST (EST-SSR) libraries [13-16], while the use of SNPs has started to be reported more recently [17-22].

In AM studies, population structure is commonly estimated by using SSR derived information, because of the proven usefulness of this type of markers for population genetics inferences and their higher information content when compared to biallelic markers [9,23-28]. Nowadays, the increased availability of SNP markers, and their more rapid and highly automated genotyping technologies, have motivated their utilization for diversity studies and for the evaluation of population structure [19,20]. Given the different mutational dynamics of SSR and SNP markers and the growing use of the latter for a wide range of applications in cultivated species, it is of interest to compare the performance of both types of markers on the same set of individuals, to evaluate if the measures of population structure and genetic diversity in sunflower are affected by the marker type of choice as it was reported for other crop species [26,27].

Here we present the genetic characterization of the 137 inbred lines that currently compose the INTA association mapping population (AMP-IL), and of a set of allied open-pollinated (OP) and composite populations (CP). The aims of this study were: (a) to assess the levels of molecular diversity and population structure using gSSR, EST-SSR and SNP; and (b) to compare the performance and the estimates produced by the different types of markers.

## Results

### Assessment of genetic diversity using SSR markers

A total of 170 sunflower accessions, corresponding to the AMP-IL (137 accessions), and a set of CP and OP (33 accessions) were analyzed using 42 SSR markers. Missing data accounted for 4.57% of the data matrix. For the full panel of accessions, the probability of identity (PI) was 3.5 × 10^−27^, the probability of identity among siblings (PI_sibs_ ) was 3.3 × 10^−12^, and the average Polymorphism Information Content (PIC) was 0.50. In the whole collection, the total number of alleles was 208, and ranged from 2 to 14 per locus, with an average of 4.95. The expected heterozygosity (He) across the total 646 sampled plants was 0.51 ± 0.16. Of the 208 alleles present in the sunflower accessions, 10 were private, or unique to the AMP-IL. In contrast, 36 private alleles were detected for the OP + CP group. The AMP-IL and OP + CP collection had 162 alleles in common. Within the AMP-IL, 25 alleles were unique to the maintainer (HA) lines, while 16 were private to the restorer (RHA) lines; and 36 alleles were private to the Argentinean germplasm when compared to the “Other origin” germplasm.

Variability indices obtained from EST-SSR were always lower than those derived from genomic SSR (gSSR). Genetic diversity statistics for each of the SSR and cultivar categories used in this study are presented in Table 1.

**Table 1:** **Summary statistics of genetic variation for the sunflower accessions included in this study using SSR markers**

**Markers**	**Sample**	**N acc.**	**N ind.**	**A**	**a**	**He**	**Ho**
All SSR	Total	169	646	208	4.95 ± 2.60	0.51 ± 0.17	0.06 ± 0.04
	OP + CP	33	235	198	4.71 ± 2.50	0.52 ± 0.16	0.09 ± 0.06
	AMP –IL	137	411	172	4.09 ± 2.16	0.48 ± 0.17	0.01 ± 0.03
	HA	59	177	156	3.71 ± 1.91	0.44 ± 0.19	0.01 ± 0.03
	RHA	78	234	147	3.50 ± 1.76	0.46 ± 0.16	0.01 ± 0.03
	Argentinean	104	312	167	3.97 ± 2.16	0.47 ± 0.17	0.01 ± 0.03
	Other origin	33	99	136	3.24 ± 1.69	0.47 ± 0.19	0.01 ± 0.03
gSSR	Total	169	646	121	5.50 ± 3.20	0.56 ± 0.14	0.07 ± 0.03
	OP + CP	33	235	113	5.14 ± 3.06	0.56 ± 0.13	0.10 ± 0.05
	AMP –IL	137	411	102	4.63 ± 2.68	0.53 ± 0.01	0.00 ± 0.00
	HA	59	177	89	4.05 ± 2.36	0.48 ± 0.19	0.00 ± 0.01
	RHA	78	234	85	3.86 ± 2.16	0.51 ± 0.13	0.00 ± 0.00
	Argentinean	104	312	99	4.50 ± 2.68	0.52 ± 0.14	0.00 ± 0.00
	Other origin	33	99	78	3.55 ± 2.11	0.54 ± 0.14	0.00 ± 0.01
EST-SSR	Total	169	646	87	4.35 ± 1.60	0.46 ± 0.19	0.05 ± 0.05
	OP + CP	33	235	85	4.25 ± 1.65	0.47 ± 0.18	0.08 ± 0.06
	AMP –IL	137	411	70	3.50 ± 1.19	0.42 ± 0.19	0.02 ± 0.04
	HA	59	177	67	3.35 ± 1.22	0.39 ± 0.19	0.02 ± 0.03
	RHA	78	234	62	3.10 ± 1.07	0.41 ± 0.18	0.03 ± 0.04
	Argentinean	104	312	68	3.40 ± 1.19	0.42 ± 0.18	0.02 ± 0.04
	Other origin	33	99	58	2.90 ± 1.02	0.40 ± 0.20	0.03 ± 0.04

N acc.: Number of sunflower accessions; N ind.: Number of individuals analyzed; A: number of alleles; a: mean number of alleles per locus; He: unbiased expected heterozigosity, Ho: observed heterozigosity. Sunflower accessions were grouped according to the categories described in the Methods section**.**

### SNP diversity in the AMP-IL

**Figure 1:**
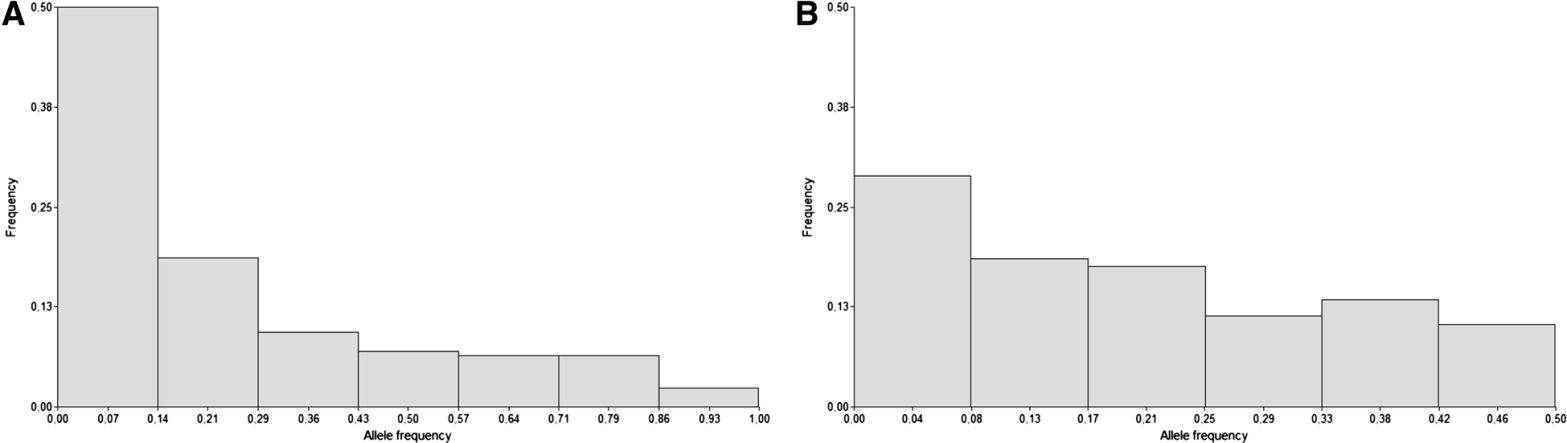
**Allele frequency distributions. A**. For the 42 SSRs. **B**. For the 182 SNPs.

The AMP-IL was further characterized using a 384 Illumina SNP-oligo pool array. Markers were removed from the data set if they were either monomorphic (80/384 markers), showed more than 10% missing values or had ambiguous SNP calling. The resulting data set was composed of 182 high quality informative SNPs. The average proportion of missing data was 0.91%. The PI was 1.0 × 10^−46^ and the PI_sibs_ was 3.3 × 10^−24^. The estimated PIC was 0.232. Inspection of the distribution of SNP allele frequencies showed a pattern different from that observed for SSR, with a larger proportion of alleles at intermediate frequencies (Figure 1).

Minor allele frequencies (MAF) were larger than 0.1 for 91.76% of the 182 polymorphic SNP loci. Overall, the expected heterozygosis (He = 0.29) was lower than the values observed for SSR markers. As expected for inbred lines, the observed heterozygosis was very low for both the SSR and the SNP data sets (0.01 and 0.03, respectively). Diversity indices obtained from SNP markers are summarized in Table 2.

**Table 2:** **Summary statistics of genetic variation for the INTA sunflower association mapping population using SNP markers**

**Sample**	**N acc**	**A**	**a**	**He**	**Ho**
AMP –IL	137	364	2.00 ± 0.00	0.29 ± 0.17	0.03 ± 0.03
HA	59	354	1.95 ± 0.23	0.29 ± 0.17	0.02 ± 0.03
RHA	78	347	1.90 ± 0.29	0.27 ± 0.17	0.03 ± 0.03
Argentinean	104	359	1.97 ± 0.16	0.28 ± 0.17	0.03 ± 0.03
Other origin	33	349	1.92 ± 0.27	0.28 ± 0.17	0.03 ± 0.03

N acc.: Number of sunflower accessions; A: number of alleles; a: mean number of alleles per locus; He: unbiased expected heterozigosity, Ho: observed heterozigosity. Sunflower accessions were grouped according to the categories described in the Methods section.

### Population structure

Analysis of molecular variance (AMOVA) was conducted to test putative differences among the groups defined in the present work (the AMP-IL vs. the OP + CP) and between the subgroups in which the AMP-IL was further subdivided (“HA” vs. “RHA”, “Argentinean” vs. “Other Origin”). Significant differences were detected between AMP-IL and OP + CP (42 SSR, FST = 0.025, p < 0.001). Within the AMP-IL, the analyses were done using the three marker-sets available: SSR, SNP and SSR + SNP. In all three cases, the AMOVA revealed significant differentiation among the groups delimited within the AMP-IL; however they only explained 2-3% of the total variance, with the remaining variation resting among individuals within groups and within individuals (Additional file 1).

Population structure estimation for the whole panel of accessions, including the AMP-IL, the OP and the CP, was done using the Bayesian clustering approach implemented in STRUCTURE. Given that the log likelihood values increased progressively as K increased, the method of Evanno et al. [29] was applied as a criterion to infer the most likely K value. The maximum delta K was detected at K = 2 with a second maximum at K = 5. Although there was a clear signal of population structure, the optimal value of K was difficult to determine since no single unifying characteristic was apparent for any of the inferred groups either at K = 2 or K = 5 (Additional file 2: Figure S1 A and B). Inspection of the DAPC plot also revealed the presence of genetic structure within these accessions (Additional file 2: Figure S1 C). The sequential k-means algorithm identified 14 groups, and the eigenvalues of the analysis showed that the genetic structure was captured by the first three PCs. As in the case of Bayesian clustering, no clear associations between the groups retrieved from DAPC and morphological, phenological or agronomical traits were found (*e.g.*, branching pattern, days to flowering, disease resistance profile, oil content).

**Figure 2:**
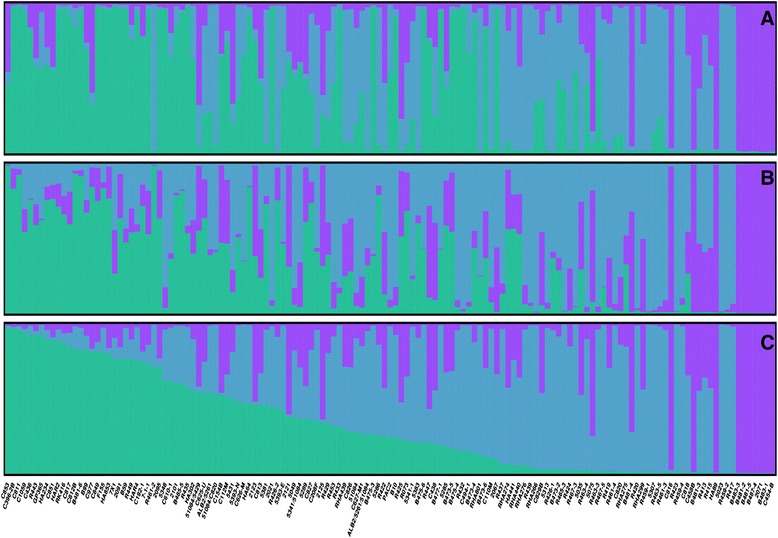
**Results of STRUCTURE for K = 3. A**. Population structure in the AMP-IL assessed with SSR. **B**. Population structure in the AMP-IL assessed with SNP. **C**. Population structure in the AMP-IL assessed with SSR + SNP.

To test the performance of the different marker sets (SSRs, SNPs and SSRs + SNPs) for predicting population STRUCTURE, the AMP-IL was subjected to further analysis. The method of Evanno et al. [29] detected three deltaK peaks at K = 2, K = 3, K = 5, for SSR and SNP data; and at K = 2, K = 3, K = 5 for the SSR + SNP data set, with the sharpest peak at K = 2 for both SNP and SSR + SNP data sets; and at K = 3 for the SSR data set (Additional file 3). Given that deltaK peaks at K = 2 have been suggested to be artefactual [30] and that all three datasets showed peaks at K = 3, graphical representation of population structure was based on K = 3 (Figure 2). The percentage of individuals assigned to a given population, i.e. with inferred ancestry >0.70, was lower for the SNPs than for the other two marker sets irrespective of the K-value being considered (Table 3). Groups 1 (Violet) and 3 (Green) are mainly composed of maintainer lines, whereas restorer lines are mostly clustered into group 2 (Light blue). Allele frequency divergence between STRUCTURE gene pools ranged from 0.13 to 0.16 between groups 1 and 2, from 0.12 to 0.14 between groups 1 and 3 and from 0.06 to 0.09 between groups 2 and 3, depending on the data set considered.

**Table 3:** **Percentage of individuals assigned to STRUCTURE populations (inferred ancestry >0.70)**

**Markers**	**k = 2**	**k = 3**	**k = 4**	**k = 5**
SSRs	83.94	67.88	**-**	69.34
SNPs	67.88	52.55	-	48.91
SSR + SNP	77.37	57.66	59.12	-

**Figure 3:**
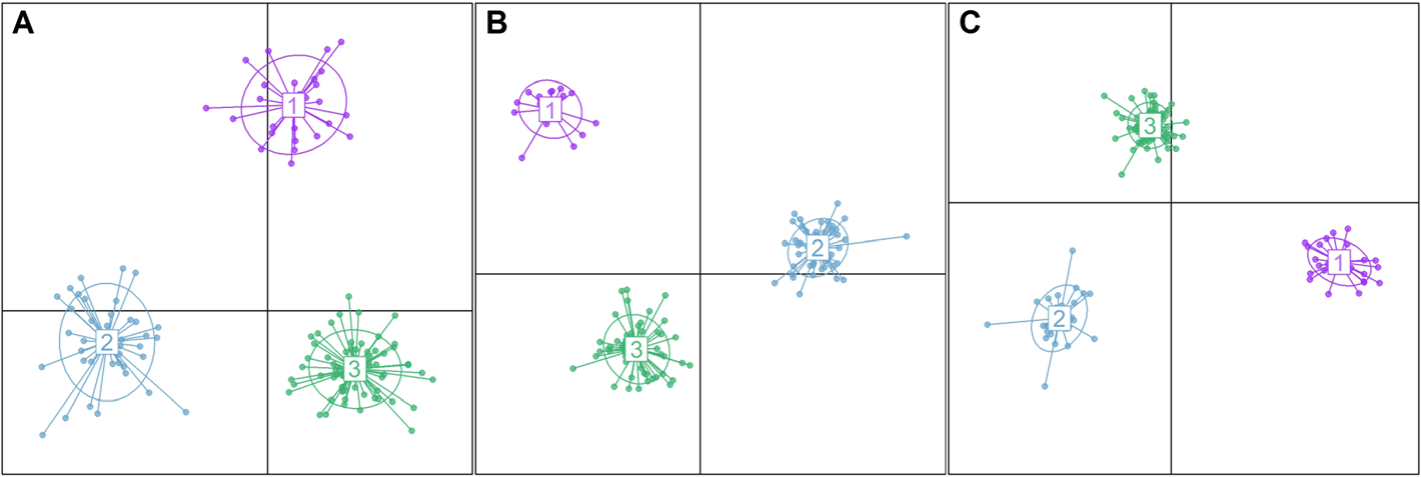
**Scatter plots of DAPC showing the first two principal components. A**. SSR data set. **B**. SNP data set. **C**. SSR + SNP data set.

Inspection of the DAPC plot also revealed the presence of genetic structure within the AMP-IL. In agreement with the STRUCTURE analysis, the sequential k-means algorithm identified 3 groups regardless of the data set under study (Figure 3, Additional file 4).

To test the consistency of individual assignments across marker sets, we computed Spearman correlation coefficients between STRUCTURE membership coefficients. Correlations between SSR and SNP outputs were significant for all three groups (r G1 = 0.6; r G2 = 0.51 and r G3 = 0.49; p < 0.0001, respectively). Significant correlations were also found when comparing SSR vs SSR + SNP (r G1 = 0.7; r G2 = 0.65 and r G3 = 0.68; p < 0.0001, respectively) and SNP vs SSR + SNP (r G1 = 0.93; r G2 = 0.83 and r G3 = 0.82; p < 0.0001, respectively).

To assess the correspondence among the groupings retrieved under Bayesian and multivariate approaches, we computed the percentage of individuals assigned to STRUCTURE groups that were assigned to the same group using DAPC. As shown in Table 4, the groups delimited by both methods were largely concordant.

**Table 4:** **Percentage of individuals assigned to the same group using STRUCTURE and DAPC**

	**SSR**			**SNP**			**SSR + SNP**		
**STR**	Group 1 STR	Group 2 STR	Group 3 STR	Group 1 STR	Group 2 STR	Group 3 STR	Group 1 STR	Group 2 STR	Group 3 STR
**DAPC**	100	79.48	97.56	100	53.84	86.36	100	63.16	88.88

STR: Structure.

**Figure 4:**
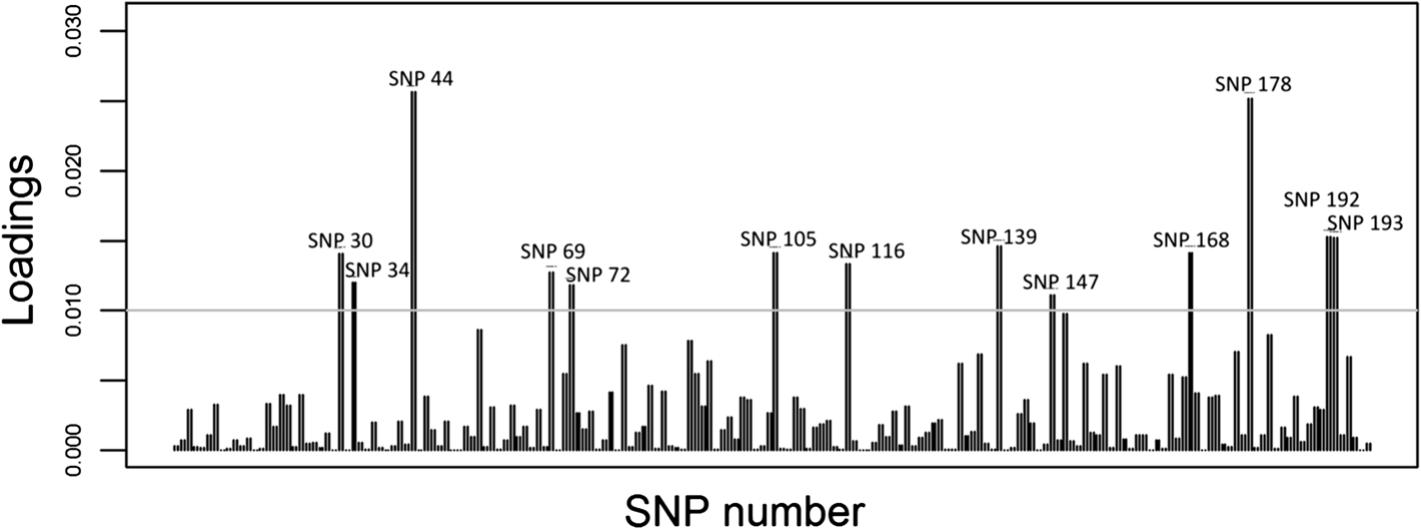
**Contribution of SNP alleles to DAPC among-group differentiation within the AMP-IL.** The height of each bar is proportional to the contribution of the corresponding allele. Only alleles whose contributions are above an arbitrary threshold of 0.010 (grey horizontal line) are indicated.

One interesting aspect of the DAPC method is that it allows the identification of those alleles that are most relevant to group delimitation. To get some insight into the underlying causes of the differentiation among the groups detected within the AMP-IL, we inspected the associated allele loadings for the SNP dataset, since it was the only set for which functional annotation was available. A total of 13 SNP were identified as the most contributing: SNP 30, 34, 44, 69, 72, 105, 116, 168, 178, 192, 193 (both alleles) and SNP 139 and 147 (1 allele). The loading plots for each type of marker are presented in Figure 4. When analyzing the gene ontology (GO) annotations associated to each marker, seven of them were related to the metabolic process category.

Distance matrices based on allele sharing were constructed for all pairs of individuals using either SSR or SNP data. For the SSR data set, distances varied from 0.012 to 0.78, with an average of 0.47. For the SNP dataset distances ranged from 0.003 and 0.45, with an average of 0.28. The Neighbor-joining trees depicting the relationships among inbred lines are provided in Additional file 5. A significant correlation was observed between the genetic distance estimates based on SNPs and SSRs, as determined by the Spearman correlation coefficient (r = 0.419; Mantel test p < 0.05).

## Discussion

Argentina has a long tradition of sunflower breeding, and its germplasm is a valuable genetic resource worldwide, with several international differential lines being derived from Argentinean varieties [3,31].

The inbred lines included in the present work were part of the first association mapping study reported for sunflower [9] and are an essential component of the INTA sunflower breeding program, as different complex characters are currently being assessed on these accessions. We also included a selection of allied OP and CP in the analysis in order to compare the levels of genetic diversity contained within the AM panel to the potential diversity present in the germplasm preserved at the Active Germplasm Bank, INTA Manfredi (AGB-IM).

In most studies, population structure and genetic diversity are commonly estimated using SSR derived information. Genomic SSR (gSSR) are attractive markers for population diversity studies because of their abundance, reproducibility and high levels of polymorphism. Recently, there was an increase in the use of EST-derived SSR, as they can be easily obtained by electronic search of EST databases. These kinds of markers that belong to the coding regions of DNA are expected to be more conserved than gSSR. Nevertheless, only few studies documented the difference in information content and other estimates of genetic variation [32-35]. Comparison between gSSR and EST-SSR in the full sunflower panel and within the AMP-IL panel confirms that gSSR markers are able to capture higher levels of diversity than EST-SSR (measured as total number of alleles, He and PIC). These results are consistent with those obtained by Hu et al. [33] in the evaluation of gSSR and EST-SSR markers for estimating genetic diversity in other non-model species, such as cucumber.

All the SSR markers selected for this analysis were successfully amplified in the whole panel of sunflower accessions. In the case of SNPs, 68% of the loci represented in the Illumina array could be successfully scored in our sample of accessions. The failure of the remaining 32% may be attributed to the origin of the SNPs included in this array, as they were discovered by *in silico* searches from EST databases [18]. According to Wang et al. [36] and Lepoittevin et al. [37], genotyping failures in ESTs-derived SNPs are common, being the result of sequence errors and consequent false-positive SNP identification, low quality of SNPs flanking sequences, or the existence of an exon-intron junction in the proximities of the selected SNP. Nevertheless, the percentage of good quality SNPs attained here is not low, when compared to other SNP panels developed for non-model species through i*n silico* approaches (42% in maritime pine [37] and 66.1% in Eucalyptus [38]).

The analysis of polymorphism in the set of sunflower accessions tested here showed that both the microsatellites and SNP markers were informative, although to different extents. To test the discriminant capacity of the panel of markers, PI and PI_sibs_ were estimated. Within the AMP-IL, the PI for the 42 SSR loci was 3.5× 10^−27^, and PI_sibs_ = 3.3 10^−12^. For the 182 polymorphic SNPs, the PI was 1.0 10^−46^, and PI_sibs_ = 3.3 10^−24^, suggesting that both panels of markers have a high discriminant capacity for sunflower germplasm collections, with the SNP data set being the most informative. Yu et al. [39] suggested that over 10 times more SNPs than SSRs should be used, while Van Inghelandt et al. [40] proposed a range between 7 and 11 times. In the present study, a total of 109 randomly chosen SNPs were enough to reach the same PI as the 42 SSR markers, suggesting that even though a higher number of SNPs are required to obtain the same information content of SSR markers [27,39,40], the ratio of the number of SNPs to SSR is strongly dependent on the characteristics of the markers and the species being considered.

According to theoretical expectations, the distribution of allele frequencies differed between SNPs and SSR markers. There was a higher presence of SSR alleles at low frequencies, whereas SNPs showed more alleles at intermediate frequencies. These spectra of allele frequencies are consistent with previous studies [26,27,41], since SSRs are commonly dominated by rare alleles.

Different mutational processes govern allelic variation at SSR and SNP loci, with mutation rates of SNP being several orders of magnitude lower than those of SSR. As a consequence, SNPs are typically biallelic, whereas SSR generally have high allelic richness and heterozygosity levels [42]. The gap between He estimates from SSR and SNP data found in this study (0.52 and 0.29, respectively) seems to be smaller than the breach observed in other crop species, such as grape (0.81-0.34) [43], maize (0.80-0.32) [27], and soybean (0.77- 0.35) [44]. These differences are mostly caused by the relatively low He estimates obtained here for SSR data, which might have been underestimated as a consequence of including EST-SSR markers.

Contradictory results have been reported by different studies regarding the correlation of genetic distances estimated with SSR and SNP markers. Jones et al. [45] and Hamblin et al. [27] found no significant correlation between genetic distance measures in maize populations, except for closely related individuals, whereas significant correlations were observed by Wurschum et al. [26] in wheat, irrespective of the range of distances being considered. In the present work we observed a significant correlation between genetic distances derived from SSR and SNP markers, suggesting that both marker types are equally appropriate to survey and classify genetic variation in sunflower.

In general, the estimates of genetic diversity obtained here for the AMP-IL are moderate and slightly lower than those detected in the 271 NCRPIS and INRA lines that compose the association mapping population used by Mandel et al. [12,19]. It should be bared in mind, however, that comparison of diversity indices is not straightforward given the differences in the number of inbred lines analyzed in each case and the fact that only one confectionary sunflower inbred is currently included in the INTA AMP-IL.

Analysis of diversity levels in the full sunflower panel and the AMP-IL showed that the latter did not comprise all the alleles that are present in the OP and CP. This suggests that new inbred lines could be included in our AM panel to fully capture the allelic diversity preserved at the AGB-IM. This reduction or apparent loss of genetic diversity is a common consequence of the sampling strategy, where the alleles in lower frequency are less likely to be captured. Similarly, the AM panel used by Mandel et al. [19] did not include all the alleles detected in NCRPIS and INRA collections from which it was derived [12].

Differences in the number of alleles and the number of private alleles were detected among the categories in which the AMP-IL was subdivided, but interestingly, there were no detectable differences in terms of expected heterozygosity. Several studies have evaluated the levels and distribution of genetic diversity in different sunflower accessions [2,12,46]. In agreement with our findings, Mandel et al. [12] found no detectable differences in allelic diversity among the different categories in which their cultivated sunflower pool was subdivided (*e.g.,* HA, RHA, Oil, Non-Oil).

The occurrence of population genetic structure was evaluated via analysis of molecular variance (AMOVA). Although moderate, significant structuring of genetic variation was found between the AMP-IL and the group composed of OP + CP and also between the different classes in which the AMP-IL was subdivided, confirming the previously suggested differences between Argentinean germplasm and that from other origins (*e.g.*, Russia, Israel, Europe, USA).

In addition, two separate methods with different statistical basis were used here to identify genetic groups and perform individual assignment, *i.e.* STRUCTURE [47] and DAPC [48]. STRUCTURE is widely used for identifying population subdivision, but it was developed for natural outcrossing populations and has the assumption of Hardy-Weinberg equilibrium which is violated by most breeding materials, including inbred lines. DAPC can thus be regarded as a more valid method for AM panels, because it relaxes the assumption of Hardy-Weinberg equilibrium [48].

Evidence of genetic substructure within the AMP-IL was found consistently for all data sources (*i.e*. SSR, SNP and SSR + SNP). Under the Bayesian approach, the results were almost identical regardless of the data set. However, we observed that far more individuals were classified as mixed when using the SNP data than when using either the SSR or SSR + SNP data sets. Moreover, as K increased, the percentage of assignment declined for the SNP data set. Differences in assignment percentages between SSR and SNP markers were also reported by several studies [27,40,41,43] and were attributed to the higher information content of SSRs [43,49].

Analysis of Spearman correlations revealed that for those individuals that exceeded our arbitrary 0.7 membership threshold group assignment was very consistent across marker sets, with group 1 being the most conserved and well defined. Indeed, inspection of the distances among STRUCTURE clusters showed that group 1 was the most differentiated.

The constitution of the three groups obtained with the DAPC approach using the different marker sets was similar, but not equal. Nevertheless, by considering those lines that were consistently assigned to the same group regardless of the marker set, a general pattern of affiliations emerged from these analyses (Additional file 4). DAPC group 1 was mainly composed of maintainer germplasm and greatly influenced by the contribution of the public inbred line HA89, which was involved in the origin of lines 2071, 2125, C454B and B71 (Additional file 6). DAPC group 2 was dominated by the presence of restorer lines, including the public inbred lines RHA801 and RHA276. The majority of the Argentinian lines included in this group were developed as part of the INTA Drought Stress Breeding Program, with their progenitors having different contributions from wild *Helianthus* species. Finally, DAPC group 3 was the largest and most diverse, with a large proportion of maintainer lines. The lines included in this group are derived from public sources from USA and from traditional Argentinian varieties, such as Impira INTA, Sáenz Peña and RusoxKlein (Additional file 6). These lines are characterized by the contribution of Russian germplasm different from that involved in the origin of HA89.

As previously mentioned, there was a large correspondence between the DAPC groups and those generated by STRUCTURE at K = 3, although with minor differences. These discrepancies could be due to the fact that under the DAPC algorithm all the lines are classified into a group, even if some admixture is detected. This was not the case for the STRUCTURE approach, where lines were arbitrarily assigned to a group when they surpassed the membership threshold of 0.7.

In addition to the detection of genetic groups within the AMP-IL, DAPC was also used to identify those alleles with the largest contributions to the discriminant functions, as an approach to detect putative patterns among the genes responsible for group differentiation [48]. A plot of SNP allele contributions was used to identify alleles of major interest, and, remarkably most of them corresponded to genes assigned to the metabolic process GO category. Although further studies are still needed to determine whether these SNPs are directly involved in inbred differentiation or if they are in linkage disequilibrium with some other, more relevant, polymorphisms or genomic regions, these results serve to highlight the potential of the DAPC method to go beyond mere group delimitation.

While STRUCTURE and DAPC clusters generated from each data set easily separated individuals into similar groups, distance methods were less capable of identifying reproducible groups for the different data sets. When compared to the STRUCTURE results at K = 3, the three NJ phylograms generated –one for each marker set- were consistent in that STRUCTURE group 1 was again well delimited, however, the NJ tree showed almost no discernible phylogenetic structure among individuals from the remaining two groups. This is in agreement with the results reported by previous authors [50,51] and with the proposals of Rosenberg et al. [52] who argued that STRUCTURE uses individual genotypic data more efficiently than phylograms based on genetic distance matrices. Overall, the population structure patterns detected here for the INTA AMP-IL are concordant with those reported by Mandel et al. [19] and Cadic et al. [20] for the NCRPIS and INRA collections, with the maintainer/restorer status being the most prevalent characteristic associated with group delimitation. In agreement with our findings, three groups were detected by the aforementioned studies, two of them consisting of maintainer or “B” lines, and the third one composed of restorer or “R” lines. Although affiliations among the groups that were found by different authors still remain to be determined, it is interesting to note that while in both Mandel et al. [19] and Cadic et al. [20] studies the maintainer groups seem to be more closely related to each other than to that of the restorer lines, our STRUCTURE results suggest a closer relationship between the restorer group and the maintainer group 3. A similar, albeit not so clear, pattern arises from inspection of DAPC plots. In sum, it appears that the worldwide distribution of genetic diversity in cultivated sunflower follows a common pattern dominated by the restorer/maintainer status.

The extent to which a given molecular marker set is able to capture population structure may have practical, and economical, implications when having to genotype large numbers of individuals. For example, the three groups detected by Mandel et al. [19] by using ca. 5500 SNP were not identified when using 34 EST-SSR on the same set of accessions [12]. Similarly, ca. 6000 SNP were included in the analysis of the 384 inbreds of Cadic et al. [20], whereas the 136 SNP used by Talukder et al. [53] on a panel of 260 diverse inbred lines retrieved only two groups in the STRUCTURE analysis. In this respect, the 42 SSR and 182 SNP panels examined here, either used separately or in conjunction, allowed consistent clear-cut group identification. Although this discrimination capacity is clearly dependent on the set of accessions being considered, testing the potential of these marker sets on different germplasm collections may help provide an affordable genotyping alternative with high levels of resolution.

## Conclusion

The present study constitutes the first report comparing the performance of SSR and SNP markers for population genetics analysis in cultivated sunflower. Overall, we showed that both the SSR and SNP panels used here are equally appropriate for estimating genetic diversity and population structure in our sunflower association mapping population. The generated knowledge about the levels of diversity and population structure of these inbred lines is an important contribution to sunflower breeding and conservation, and serves to complete the worldwide diversity map of cultivated sunflower.

## Methods

### Plant material and molecular markers

A set of 137 sunflower inbred lines composing the INTA Association mapping panel (AMP-IL), 13 open-pollinated (OP) and 20 composite (CP) populations from the Active Germplasm bank of INTA Manfredi (AGB-IM) were included in this study. ID, Pedigree information, and origin are summarized in (Additional file 6: Table S3).

Leaves from AMP-IL, OP and CP were collected from 3-week-old plants, sampling 3, 6 and 9 individuals, respectively. Genomic DNA was isolated from 20 mg of lyophilized material using NucleoSpin Plant II kit (Macherey-Nagel, Germany) and following manufacturer’s instructions. The quality and the concentration of the genomic DNA were assessed using electrophoretic analysis and Picogreen® technology (Invitrogen, San Diego, CA). Genomic DNA was normalized to 25 ng/μL before genotyping.

All DNA samples were genotyped using 22 genomic SSR (gSSR) selected from Paniego et al. [14] and 20 EST-SSR chosen from Chapman et al. [54] and Mandel et al. [12], resulting in at least two markers on each one of the 17 sunflower linkage groups. The SSR markers were selected based on presumptive neutrality and genetic map position, while the EST-SSR were selected for comparison of our population diversity results with those reported by Mandel et al. [12]. A list of the SSR markers included in the present study is shown in (Additional file 7: Table S4).

For further genetic characterization, the 137 AMP-IL were also examined using a custom-designed 384 SNP Illumina Oligo Pool Assay (OPA) [18,55].

SSR genotyping was performed using multiplexed PCR with fluorescent labeled primers (HEX; NED and FAM). Fragment analysis was carried out with GeneMapper® 4.0 software (Applied Biosystems, Foster City, USA) using a commercial size standard for allele size assignment (GeneScan ROX 500, Applied Biosystems®). Automatic allele calls were subsequently confirmed manually reviewing all electropherograms. Genotyping of the SNPs was performed on the Illumina GoldenGate, BeadXpress (Illumina, San Diego, CA) at the Biotechnology Institute (CICVyA, INTA) with the protocol provided by Illumina [56]. Data were analyzed using the Illumina software GenomeStudio (Illumina, Inc., San Diego, CA).

### Genetic diversity analysis

Measures of genetic diversity, including total number of alleles, mean number of alleles per locus (A), unbiased expected heterozigosity (He) [57], observed heterozigosity (Ho) and polymorphism information content (PIC) were estimated from the SSR and SNP datasets, respectively, using PowerMarker v. 3.51 [58]. For these analyses, the AMP-IL was further subdivided into different categories based on geographical origin (Argentinean or Other) and breeding history (HA: maintainer; RHA: restorer).

The probability of identity (PI), the PI considering genetic similarity among siblings (PIsibs) and the minor allele frequency (MAF) were calculated using GenAlEx [59].

### Population structure

Population structure was investigated via analysis of molecular variance (AMOVA; [60]), using GenAlEx [59]. The extent of differentiation between the AMP-IL and CP + OP was estimated using only the 42 SSR data, as the OP + CP group was not genotyped with the Illumina OPA. Analysis amongst the categories in which the AMP-IL was subdivided was carried on considering the SSR, SNP and SSR + SNP data sets. In all cases statistical significance was evaluated by doing 999 permutations.

The model-based approach implemented in the software package STRUCTURE [47] was used to infer population structure. For the SSR markers, the AMP-IL, CP and OP were first evaluated together, followed by a separate analysis of the AMP-IL. Population structure of the AMP-IL was additionally assessed using the SNP and SNP + SSR datasets. For each analysis, different population genetic clusters (K = 1–20) were evaluated with 5 runs per K value. For each run, the initial burn-in period was set to 500,000 with 500,000 MCMC iterations, under the admixture model and independent allele frequencies, with no prior information on the origin of individuals [61]. To determine the most probable value of K, the deltaK method described by Evanno et al. [29] was used as implemented in Structure Havester [62]. STRUCTURE results were displayed with the software Distruct [63]. Spearman correlations between the different groups identified using STRUCTURE were computed using the software Infostat [64].

Genetic relationships among the AMP-IL were also examined by applying the discriminant analysis of principal components (DAPC; [48]) on the SSR, SNP and SNP + SSR datasets using the Adegenet package [65] for R 3.0.2 software (R development Core Team [66]). The function DAPC was executed using the clusters identified by K-means (Legendre and Legendre [67]). The number of clusters was assessed using the function ‘find.clusters’ , evaluating a range from 1 to 40. The optimal number of clusters was chosen on the basis of the lowest associated Bayesian information criterion (BIC). Contribution of individual alleles to population structure was also estimated using the Adegenet package [65], and the function ‘loadingplot’.

Measures of genetic distance, estimated from the proportion of shared alleles, were obtained for the SSR and SNP datasets, respectively. Correlations between distance matrices were assessed using the Mantel test as implemented in GenAlEx [59].

In addition, a neighbor-joining tree was constructed based on the genetic distances calculated between pairs of accessions. Cluster analyses and bootstrap resampling (1000 pseudo replicates) were performed using PowerMarker 3.25 [58]. Branch support percentages were computed using the Consense algorithm included in the computer software package PHYLIP v. 3.68 [68].The program FigTree v. 1.3.1 [69] was then used to visualize and edit the resulting tree.

### Availability of supporting data

The source of the SNPs used for the analyses presented here is given in Additional file 8. SSR and SNP genotypes for the sunflower accessions included in this study are provided in Additional file 9.

## Additional files

Additional file 1:
**Results of the analysis of molecular variance (AMOVA).** Table. Additional file 2:
**Population structure assessed with SSR in the total panel of accessions A. STRUCTURE results for K = 2. B. STRUCTURE results for K = 5. C.** Scatterplot of DAPC (14 groups). The scatterplot shows the first two principal components of the DAPC. Figure. Additional file 3:
**Delta K values of STRUCTURE outputs for the AMP-IL.** **A**. SSR dataset; **B**. SNP dataset; **C**. SSR + SNP dataset. Figure. Additional file 4:
**Inbred lines assigned to the three groups retrieved from the DAPC analysis.** Table. Additional file 5:
**Neighbor-Joining phylograms for the 137 AMP-IL. The genotypes are colored on the basis of the STRUCTURE analysis (K = 3).** A. SSR dataset; B. SNP dataset; C. SSR + SNP dataset. Bootstrap values are indicated beside branches. Figure. Additional file 6:
**General characteristics of the sunflower accessions included in the study.** Table. Additional file 7:
**Primer sequences location and type of analysis for the SSR markers included in the present study.**
Additional file 8:
**Source of the SNPs used for analysis.** Database name and Accession numbers of the sequences from which polymorphisms were derived are provided. Additional file 9:
**SSR and SNP genotypes for the sunflower accessions included in this study.** Missing data is indicated by “?/?”.
