# Supplementary figures and images for: Population structure and genetic diversity characterization of a sunflower association mapping population using SSR and SNP markers
Source: BMC Plant Biol. 2015 Feb 13;15:52. doi: 10.1186/s12870-014-0360-x (PMC4351844; doi:10.1186/s12870-014-0360-x)

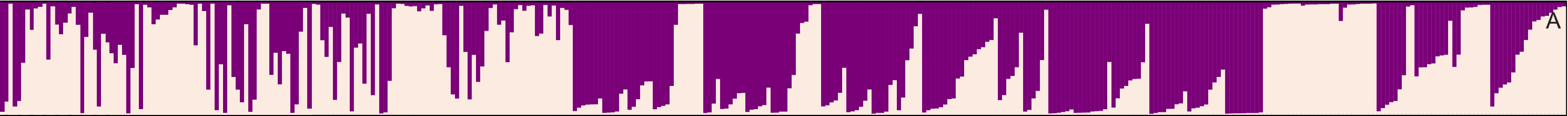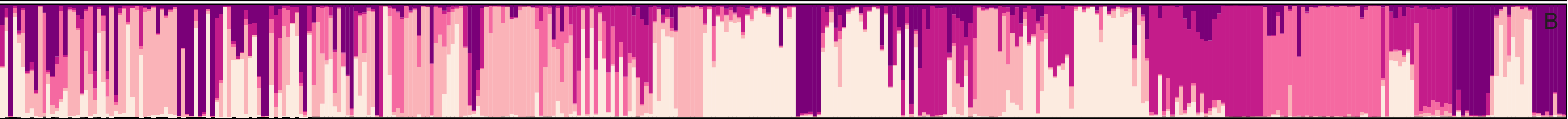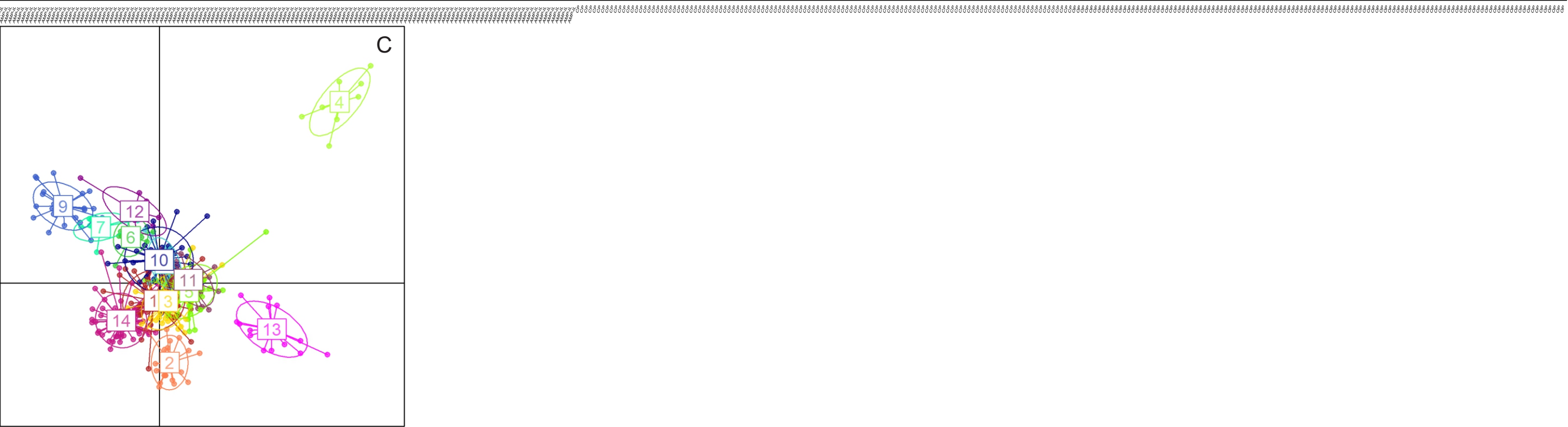

Supplement: Additional file 2: — Population structure assessed with SSR in the total panel of accessions A. STRUCTURE results for K = 2. B. STRUCTURE results for K = 5. C. Scatterplot of DAPC (14 groups). The scatterplot shows the first two principal components of the DAPC. Figure. [file 12870_2014_360_MOESM2_ESM.pdf]

A

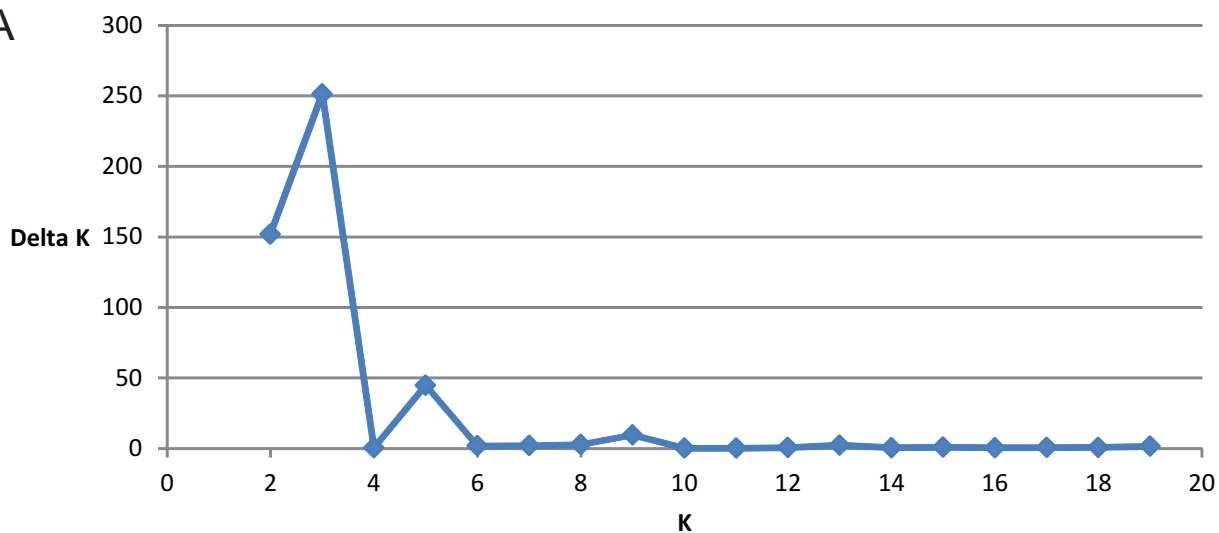

B

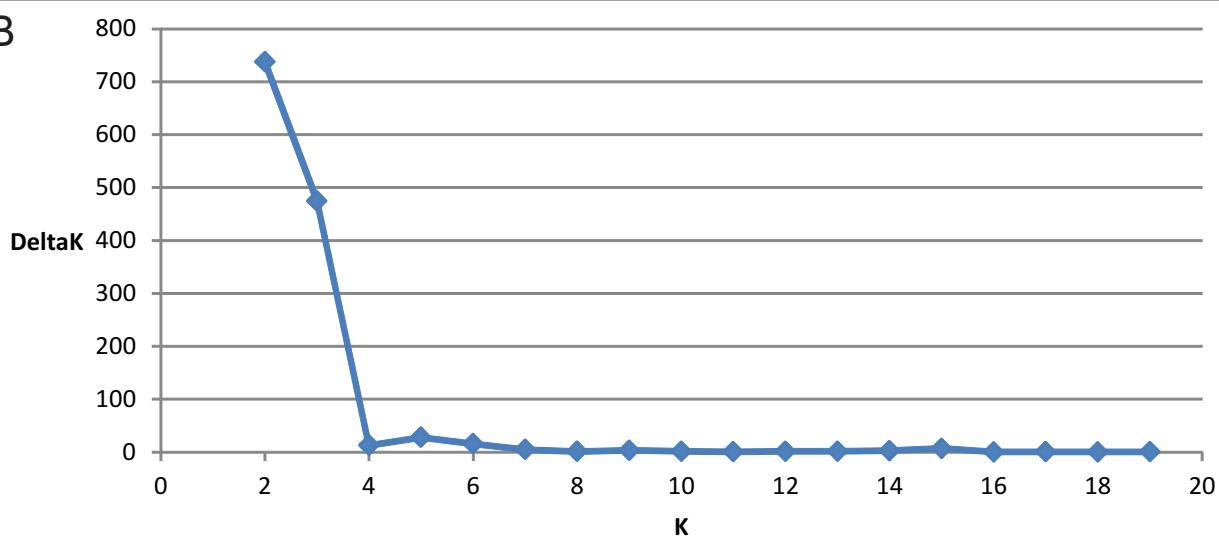

C

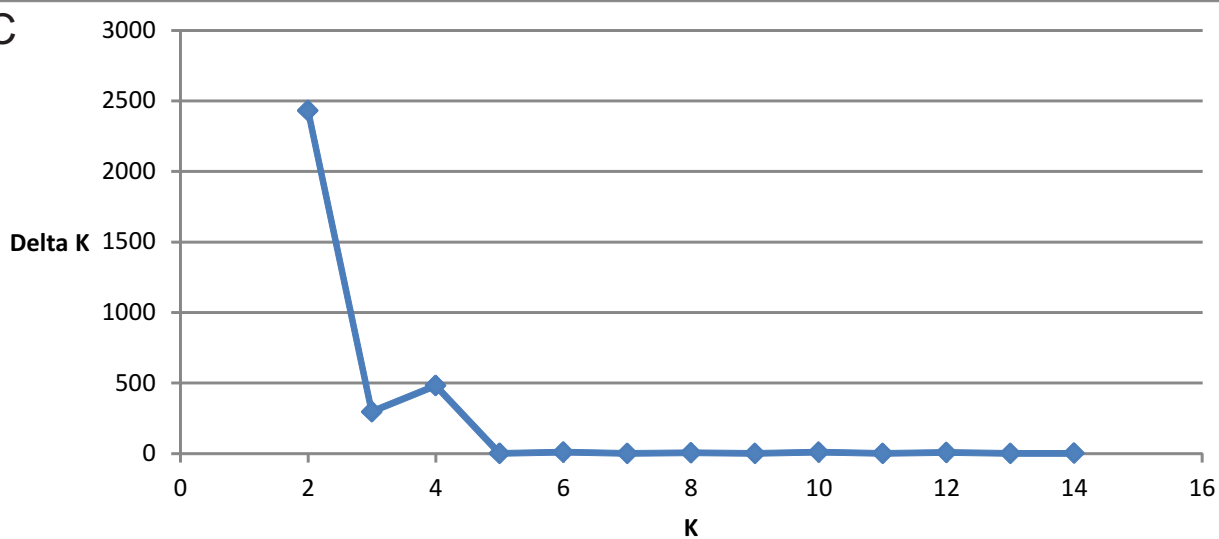

Supplement: Additional file 3: — Delta K values of STRUCTURE outputs for the AMP-IL. A. SSR dataset; B. SNP dataset; C. SSR + SNP dataset. Figure. [file 12870_2014_360_MOESM3_ESM.pdf]

A

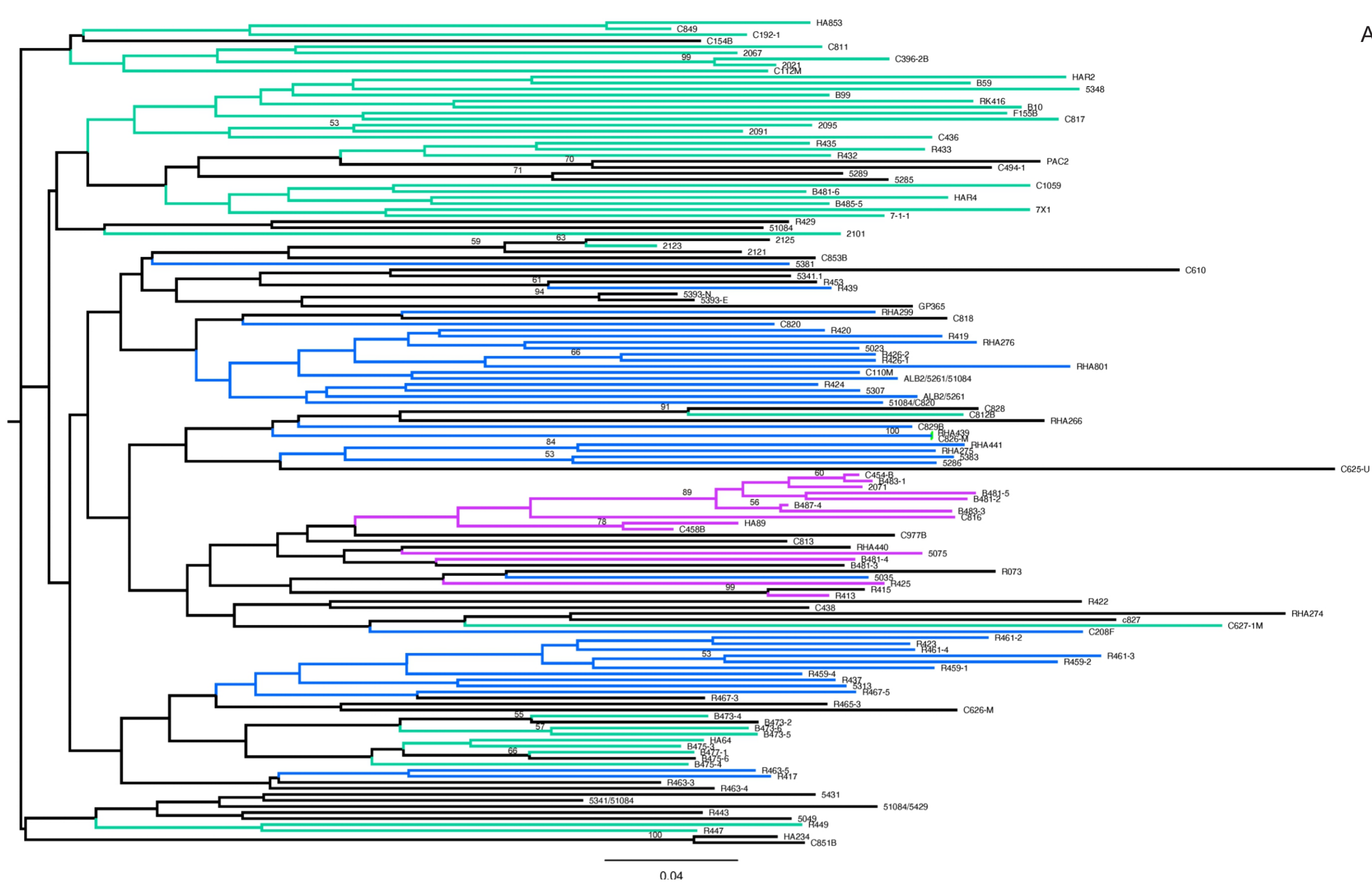

B

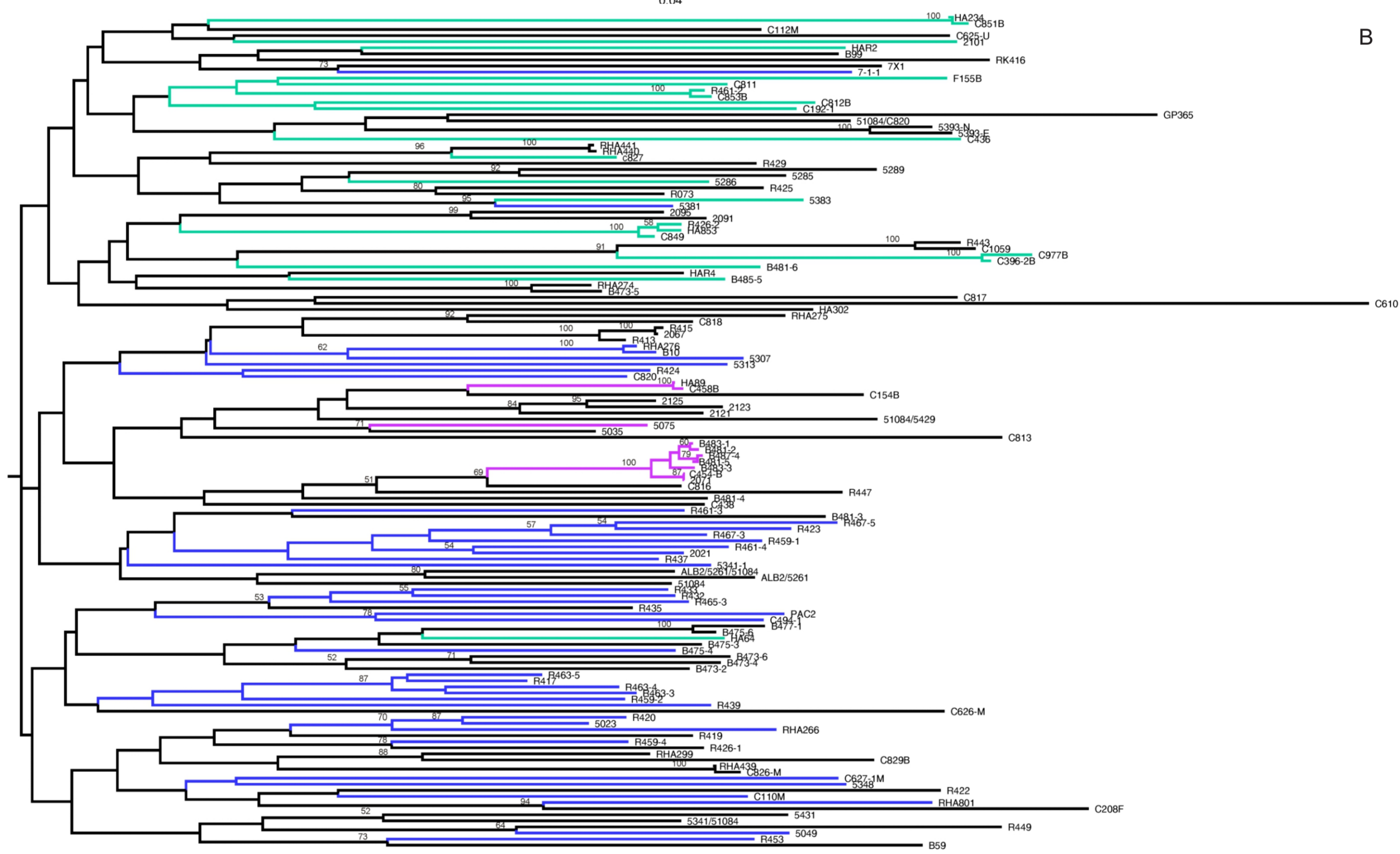

C

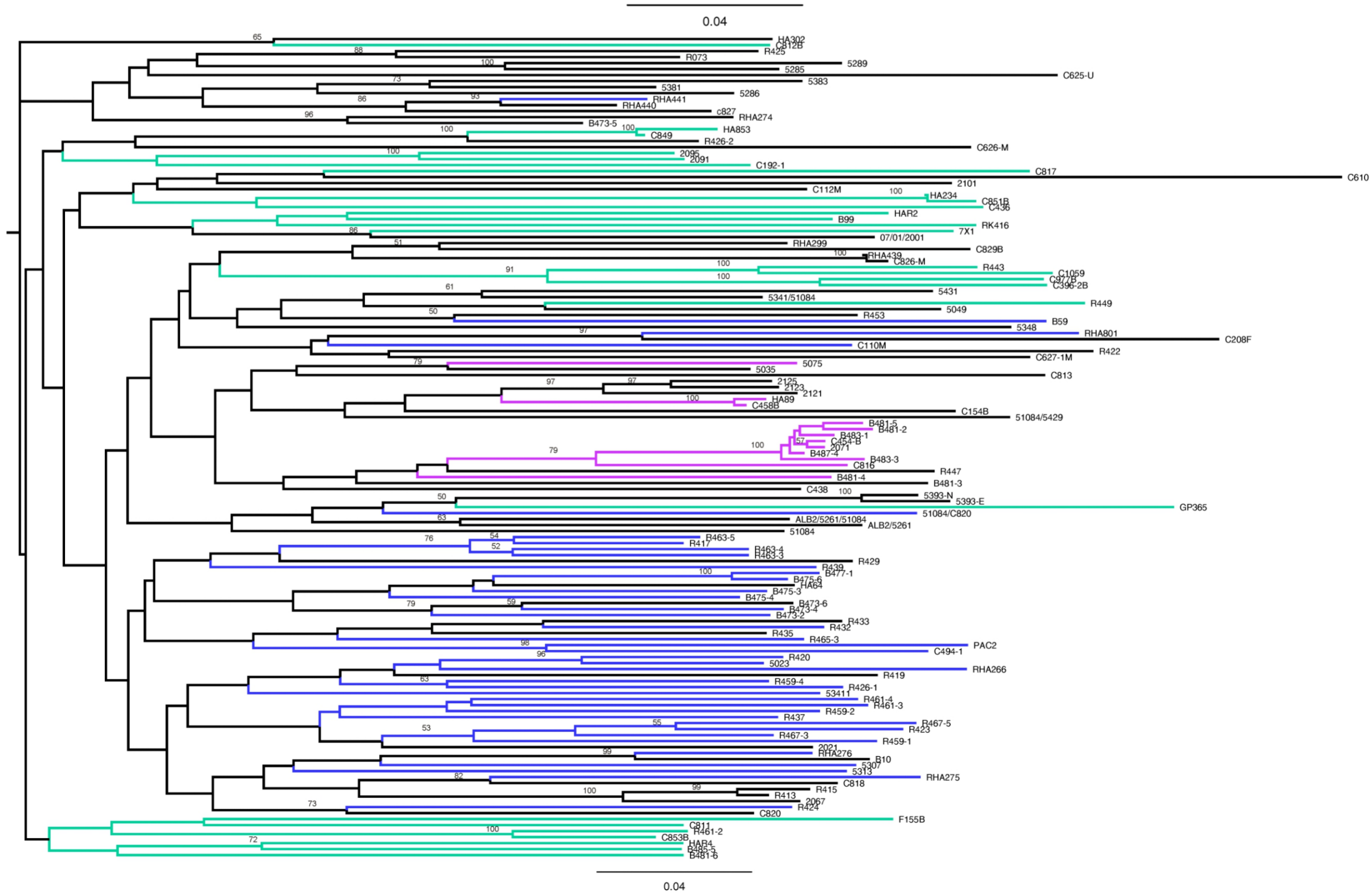

Supplement: Additional file 5: — Neighbor-Joining phylograms for the 137 AMP-IL. The genotypes are colored on the basis of the STRUCTURE analysis (K = 3). A. SSR dataset; B. SNP dataset; C. SSR + SNP dataset. Bootstrap values are indicated beside branches. Figure. [file 12870_2014_360_MOESM5_ESM.pdf]
